# Influence of sexual maturation status on the relationship between body adiposity indicators and age: a cross-sectional study

**DOI:** 10.1186/s13104-019-4095-5

**Published:** 2019-01-25

**Authors:** Livia Akemi Ramos Takahashi, Francisco Winter dos Santos Figueiredo, Jucemar Benedet, Francisco de Assis Guedes de Vasconcelos, Fernando Adami

**Affiliations:** 1 Epidemiology and Data Analysis Laboratory, Faculdade de Medicina do ABC, Santo André, Brazil; 2 Federal University of Santa Catarina, Santa Catarina, Brazil

**Keywords:** Adiposity, Obesity, Sexual maturation

## Abstract

**Objectives:**

To determine the influence of sexual maturation status on adiposity indicators of children and adolescents.

**Results:**

2412 individuals participated, 1285 (47.4%) males and 1408 (52.6%) females. There was moderate to weak correlation between age and adiposity indicators for both sexes. By analyzing the relationship between age and body fat indexes adjusted for Sexual Maturation Status, several changes were observed, mainly in girls. Precocious maturation was associated with increased adiposity indicators in girls, whereas late maturation was associated with decreased adiposity indicators in both sexes. Precocious maturation was associated with increased adiposity indicators in girls, whereas late maturation was associated with decreased adiposity indicators in both sexes.

**Electronic supplementary material:**

The online version of this article (10.1186/s13104-019-4095-5) contains supplementary material, which is available to authorized users.

## Introduction

Different sexual maturation status are related to different times of exposure to changes in the pubertal repletion and spurt phase. The body fat and skinfolds tend to increase in the final stage of puberty (pubertal repletion) [1]. And the body weight of individuals will double in adulthood while there are in the spurt of growth [2]. There are several risk factors that can lead to weight gain, such as: eating habits [3, 4], practice or lack of physical exercises [5, 6], family and genetics [7, 8], social position [9, 10], and others less studied, such as living in rural or urban areas [11] and lack of vitamin D [12], as well as the status of sexual maturation [13]. Obesity is a disease associated with various metabolic, cardiac, and neoplastic syndromes and dysfunctions, which are mainly responsible for human mortality according to the World Health Organization [14].

Overweight and obesity in children and adolescents are public health problem because they increase the risk of developing, in adulthood, several chronic diseases, such as cardiovascular, metabolic, and neoplastic diseases [14]. In addition, it is known in the literature that obesity can be associated with the stages of development: prenatal, neonatal and adolescence. In view of this, important factors that can lead to obesity are: pregnancy, breastfeeding and low social level. While the practice of physical exercises is a protective factor for overweight [5–10]. Both pubertal repletion and spurt lead to weight gain and occur during puberty because of sexual maturation, which also influences bone age (a way of describing the degree of maturation of a child’s bones), body mass, body diameter and muscle circumference [15], as well as behavioral and psychological changes, such as loss of food control [16].

Sexual maturation is an important biological marker. Its status can be classified as precocious, normal, or late. They may vary according to age and sex and are associated with both overweight and obesity in children and adolescents [17]. Given the relationship between sexual maturation, weight gain and excess weight, obesity status, and knowing that the times of exposure to the effects of sexual maturation may vary according to the maturation status; we ask if a variation can be established between in the relationship adiposity indicators and age based on the sexual maturation status in boys and girls.

To answer this question, we analyzed the correlation between age and adiposity indicators adjusted for sexual maturation status and sex of children aged 8–14 years, obtained from a database collected in 2007. Testing the hypothesis that the indicators of adiposity vary with age and sexual maturation in both sexes.

## Main text

### Materials and methods

#### Study design

This was a cross-sectional population-based study conducted following the standards for observational studies of the Strengthening the Reporting of Observational Studies in Epidemiology [18].

#### Study population

A total of 2412 individuals, 47.4% (n = 1285) males and 52.6% (n = 1408) females, were analyzed.

Data collection took place between the months of March and April 2007, in the city of Florianópolis, located in the state of Santa Catarina, southern region of Brazil.

For the selection of the sample, four schools, two public and two private, were selected in the central region of Florianópolis, where there is a concentration of almost 50% of all students enrolled in the municipality. The students were stratified according to sex and age in each school. The choice of students was made randomly.

#### Data source

Individuals eligible for the study were children of both sexes, from public and private schools, living in Florianópolis, Santa Catarina. In this place was collected the database from 2007 that we chosen to develop this research. The sampling was probabilistic, and the sample was representative of Brazilian children and adolescent population. The database was structured through the formation of a group responsible for data collection, consisting of 10 people previously trained in a workshop that took place between September 2006 and March 2007. The process of bank structuring and data reliability has been described in other studies [19, 20].

#### Ethical aspects

This project was approved by the Research Ethics Committee of the Federal University of Santa Catarina (process no. 028/066).

#### Variables analyzed in the study

The criteria used to determine the variables were:

i. Overweight:
  - Excess weight was determined using the body mass index (BMI).
  - BMI was calculated from measured body weight (Kg) and height (m2). For the measurement of weight, the individual remained in the orthostatic position (of foot, with the body erect) with the divided weight on both feet, holding the head of According to the Frankfurt Plan, shoulders relaxed and relaxed arms laterally. The child was barefoot and wearing lightweight (preferably without jeans, sweatshirts or jackets). For the measure of height the child also remained in the orthostatic position, feet together, weight divided into both feet, upper back playing the stadiometer, head according to with the Frankfurt plan, relaxed shoulders and loose arms laterally.
  - Body weight was measured to the nearest of 50 g using an electronic scale (model PP 180, Marte^®^, Minas Gerais, Brazil), capacity of 180 kg; height was measured to the nearest of 1 mm by Alturexata^®^ stadiometer.
  - For the analyses, the cut-off points for overweight by age and sex was based on the World Health Organization’s guidelines [21].
ii. Birth weight:
  - The birth weight of schoolchildren was reported by parents or guardians. Students were classified as having low weight (< 2.500 g), normal weight (≥ 2.500–3.999 g), or high weight (≥ 4.000 g) [14].
iii. Overweight mother:
  - Overweight mothers (≥ 25 kg/m^2^) of the children were classified following the recommendations provided by the World Health Organization, also according to BMI.
iv. Classification of sexual maturation:
  - In order to classify the sexual maturation, we used the criteria established by Tanner et al. [22].

#### Body adiposity

Body adiposity was assessed using the following indicators: (i) arm circumference; (ii) triceps skinfold; (iii) calf skinfolds, and (iv) total skinfolds. Triceps and calf skinfolds and arm circumference were individually analyzed as indicators of peripheral fat and the total of the four folds as an indicator of general adiposity.

Adiposity indicators evaluated by adipometer and cirtometry, respectively. A manual adipometer was used to evaluate body fat (skinfolds). The cirtometry consists of a set of measures of the circumferences of chest and abdomen during the respiratory movements. A tape measure was used for such measurements. The team that collected the data participated in a pilot study and training of standardization of anthropometric measures to measure intra and interrater errors.

#### Sexual maturation status classification

To classify the sexual maturation status, we used the criteria established by Tanner [22]. The process of collecting the information was self-assessed by children and adolescents. For this, the students went to the room where the evaluation of sexual maturation would take place. In this room, there was an experienced researcher who explained how the school should proceed. At the time of choosing the stage, the assessed was left alone in the room.

Sexual maturation status was classified based on age tertiles for each stage [23]:

i. Precocious sexual maturation—individuals below the first tertile.
ii. Late sexual maturation—individuals who were above the second tertile.
iii. Normal sexual maturation—individuals between the first and second tertiles.

#### Data analyses

We used the central tendency measures (with their respective 95% confidence intervals—CI) and Rao-Scott test to analyse the qualitative variables. Median was used to describe adiposity (due to non-normality of the data—Shapiro–Wilk test; P < 0.05) based on the sexual maturation status of boys and girls, stratified by age at 6-month intervals. For quantitative variables we used Mann–Whitney test to determine the differences in some of the study variable between males (boy) and females (girls).

Interquartile regression was used to analyze the median relationship of age-adjusted adiposity indicators according to each sexual maturation status using the following equation:

$${\text{Y}}, \, \beta 0\, + \,\beta 1 {\text{x}},\quad {\text{which were estimated}}$$

Y, age-adjusted adiposity indicators; Β, the median variation of the adiposity indicator for each year; 95% CI do β, 95% CI of the median variation of the adiposity indicator for each year; r^2^, predictive capability; *P* value, probability value.

Given the complexity of the sampling process, a correction was performed for the complex sample using the svy command in Stata 11.0.

### Results

The current overweight prevalence was 34.9 (95% CI, 31.3–38.6%) and 25.0 (95% CI, 21.9–32.7%) in boys and girls, respectively (P < 0.001). Except the birth weight condition (P < 0.001), other variables did not differ between sex. The median age in both sexes was approximately 12 years (Table 1).

**Table 1:** Characteristics of children and adolescents aged 8 to 14 years

Variables studied	Boys (n = 1285)	Girls (n = 1408)	P
	Number of participants (CI 95%)		
Child’s overweight^b^	448 (402–496)	352 (308–460)	< 0.001
*Classification of sexual maturation* ^a^			0.940
Precocious	425 (375–478)	458 (403–517)	
Late	413 (364–469)	460 (425–498)	
Overweight mother^b^	388 (301–487)	456 (398–518)	0.368
*Weight at birth (g)* ^b^			< 0.001
< 2500	71 (46–105)	118 (100–141)	
> 4000	190 (166–218)	110 (77–142)	

Variables studied	Boys (n = 1285)	Girls (n = 1408)	P
	Median (CI 95%)		
Age (years)^b^	11.9 (11.7–12.1)	12.0 (11.8–12.1)	0.419

^a^Rao-Scott test for qualitative variables

^b^Mann–Whitney test for quantitative variables

**Fig. 1:**
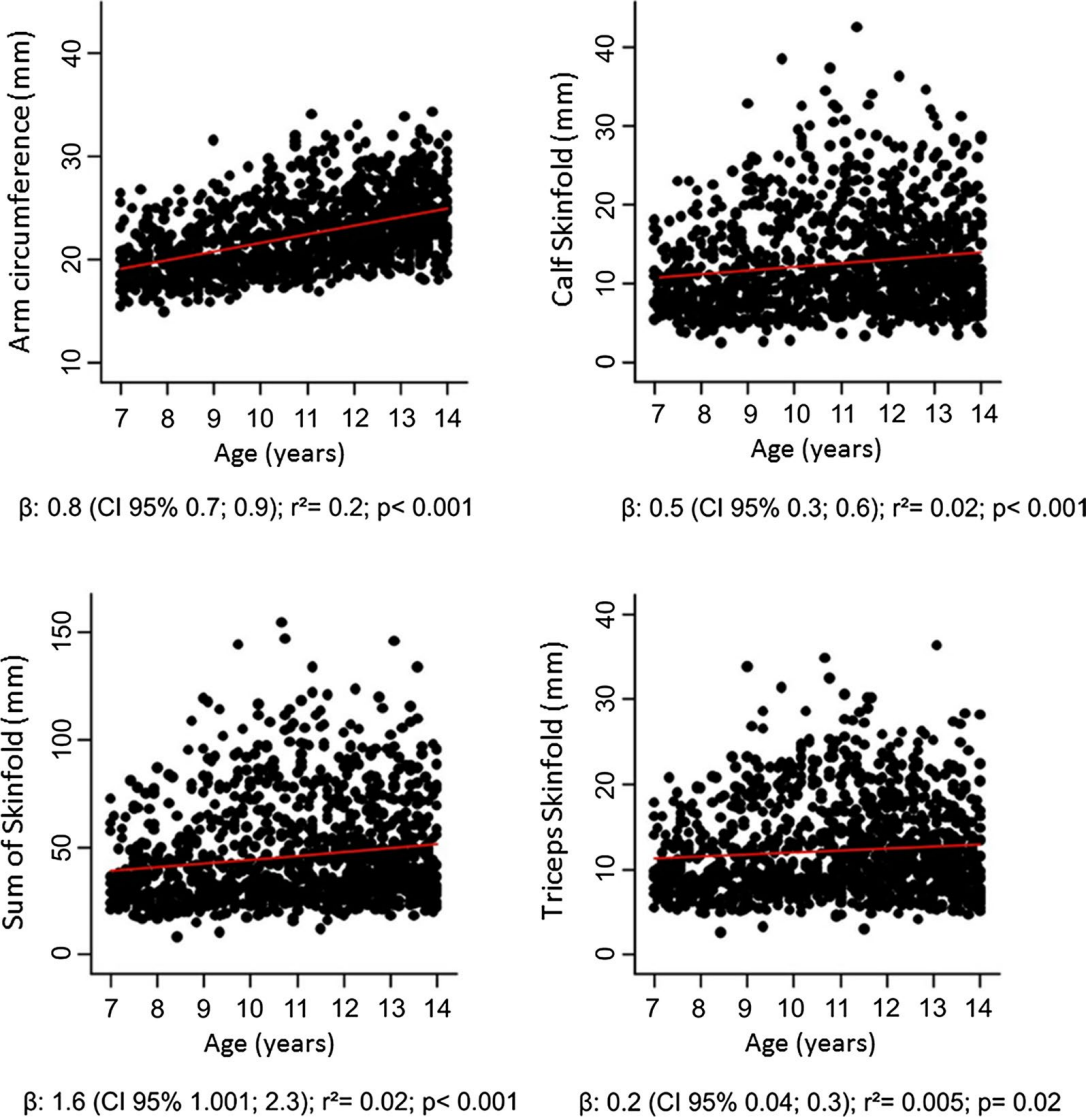
Correlation between age and body adiposity indicators in boys. Β: slope of interquartile regression

In boys, adiposity indicators increased with age, except for the triceps skinfold. Age and arm circumference (rho = 0.519; P < 0.001) were moderately correlated, whereas age and calf skinfold (rho = 0.102; P = 0.003) as well as the total of all skinfolds (rho = 0.136; P < 0.001) were weakly correlated (Fig. 1).

In girls, all adiposity indicators increased with age, as illustrated by the moderate correlation between arm circumference and age (rho = 0.527; P < 0.001) and weak correlation between age and the triceps skinfold (rho = 0.244; P < 0.001), calf skinfold (rho = 0.271; P < 0.001), and total of all skinfolds (rho = 0.314; P < 0.001) (Additional file 1: Figure S1).

When analyzing the relationship between age and adiposity indicators adjusted for the sexual maturation status, we observed several changes, mainly in girls.

In boys who matured late, a decrease of − 0.78 (ranging from − 1.39 to − 0.16) in the circumference of the arm, which was not observed in those who matured in the normal period, was found (P = 0.792). In girls, adiposity indicators in those who mature in the normal period did not change, presenting as a random finding (P > 0.05). However, all indicators significantly increased (P < 0.05) in those who mature precocious, but significantly decreased (P < 0.05) in those who mature late (Table 2).

**Table 2:** Influence of sexual maturation status on adiposity indicators in Brazilian children

Indicators of adiposity	Precocious		Normal		Late	
	β (CI 95%)	P*	β (CI 95%)	P*	β (CI 95%)	P*
*Boys*						
Triceps skinfold	0.1 (− 0.1; 0.2)	0.29	0.03 (− 0.1; 0.1)	0.62	0.1 (− 0.1; 0.3)	0.26
Arm circumference	0.46 (− 0.13; 1.05)	0.129	0.07 (− 0.43; 0.56)	0.792	− 0.78 (− 1.39; − 0.16)	0.014
Calf skinfold	− 0.01 (− 0.41; 0.39)	0.963	0.90 (− 0.17; 1.97)	0.099	− 0.92 (− 2.08; 0.24)	0.120
Sum of skin folds	1.3 (0.7; 1.8)	< 0.001	1.1 (0.6; 1.6)	0.001	1.5 (0.6; 2.3)	0.003
*Girls*						
Triceps skinfold	1.71 (1.12; 2.31)	< 0.001	− 0.08 (− 0.85; 0.69)	0.837	− 2.02 (− 2.82; − 1.22)	< 0.001
Arm circumference	1.17 (0.78; 1.55)	< 0.001	− 0.30 (− 0.67; 0.07)	0.107	− 1.20 (− 1.57; − 0.83)	< 0.001
Calf skinfold	1.57 (0.78; 2.37)	< 0.001	0.078 (− 0.80; 0.95)	0.861	− 2.43 (− 3.34; − 1.53)	< 0.001
Sum of skin folds	6.55 (4.62; 8.48)	< 0.001	− 1.53 (− 4.46; 1.40)	0.305	− 8.20 (− 11.58; − 4.82)	< 0.001

* Age-adjusted interquartile regression

When analyzing the relationship between adiposity and age indicators according to the sexual maturation and status of Brazilian children and adolescents, we found that (i) boys who matured late have a decrease in arm circumference, not found in boys who matured in the normal period and (ii) adiposity indicators increased with age in girls who matured precocious, but decreased in those who matured late.

### Discussion

Although several other studies have examined the association between sexual maturation status and obesity [15, 17, 24, 25], few studies have related sexual maturation to adiposity indicators. To our knowledge, this is one of the first studies to analyze the influence of sexual maturation status on the relationship between increased body adiposity and age in Brazilian children.

Other studies relating sexual maturation to adiposity indicators were conducted in Norway and the United States. Bratberg et al. [26], who studied 1605 adolescents in Norway, found that the waist circumference of girls with precocious sexual maturation status was associated with overweight. Staiano et al. [13], who studied 382 children and adolescents aged 7 to 16 years in the United States, found that the mammary phase of sexual maturation can influence both subcutaneous adipose and visceral tissue development in girls. According to Ferriani and Santos [1], during the growth process, a stage of pubertal repletion and spurt occurs. In repletion, body fat and skinfolds increase, while in spurt, the growth rate increases. At the end of the spurt, growth slows down and menarche ensues; and girls gain weight by increasing adipose tissue on a larger scale than boys, which is consistent with our results. Endocrine changes present in the pubertal process would be responsible for weight gain. Testosterone levels are known to be low and estradiol levels are high in obese adolescents [27]. This inverse relationship may be due to the high level of aromatase as the body weight increases. Because estradiol is related to fat and testosterone accumulation and hair development, its altered levels during puberty may explain why some studies associate obesity in children with the late appearance of pubic hair [28].

Marcovecchio [29] suggested the hypothesis that childhood obesity would promote the precocious onset of sexual maturation, reporting that during the prepubertal years, obese children would have higher growth velocity and accelerated bone age compared to lean individuals. Some studies relate leptin, produced in the adipose tissue, with the onset of puberty. However, some studies also dispute this theory by claiming that leptin does not have an ideal metabolic potential to promote puberty. Thus, Choi [30] reported that the exact primary mechanisms leading to puberty are not yet known.

In addition, we also observed that adiposity indicators tend to be higher in girls with precocious sexual maturation, but lower in those with late maturation. These findings are similar to those found in previous cross-sectional studies [15, 25] that the precocious onset of sexual maturation tend to be associated with obesity. In a study by Chipkevitch [31], girls have a higher fat deposition rate than boys, which intensifies after the peak of growth velocity during the menarche. Thus, girls have a certain propensity to gain weight with the onset of puberty, which explains the results found in the present study. All adiposity variables of the female sex were significant in precocious and late periods of sexual maturation (being that when precocious, a trend of growth indicators is obvious, which reverses when the maturation is late).

With this, we conclude that females have a natural tendency to gain weight, which intensifies over the years during menarche and precocious maturation. Although other studies agree with this finding [23]; the mechanism by which late sexual maturation leads to a tendency to reduce weight remains a gap that needs to be answered based on physiological or behavioral changes, as well as the changes found in girls.

The strengths of this study include the large sample size, larger than those in the United States [13] and Norway [26], and the adjusted analysis for complex samples on the correlation between age and adiposity indicators based on the sexual maturation status for each sex.

### Conclusion

Sexual maturation status can influence the relationship between adiposity and age indicators in girls, which increases as they mature precocious and decreases as they mature late. In boys, the late maturation status is associated with decreased triceps skinfold and age.

## Limitations

This study has some limitations as: its cross-sectional nature, which can only establish correlation, not causality. So, we are not able to say if overweight or obesity independently predict early sexual maturation. But we can affirm that these variables are correlated. Moreover, given the retrospective nature of this analysis, the data available at the time of collection were limited, and performing other analyses adjusted by other factors, such as ethnicity, is impossible [13].

### Additional file

**Additional file 1: Figure S1.** Correlation between age and body adiposity indicators in girls.

## Annex

See Table 3.

**Table 3:** STROBE statement—checklist of items that should be included in reports of *cross*-*sectional studies* [32]

	Item no.	Recommendation	Pages
Title and abstract	1	(a) Indicate the study’s design with a commonly used term in the title or the abstract	2
		(b) Provide in the abstract an informative and balanced summary of what was done and what was found	2
*Introduction*			
Background/rationale	2	Explain the scientific background and rationale for the investigation being reported	3
Objectives	3	State specific objectives, including any pre-specified hypotheses	3
*Methods*			
Study design	4	Present key elements of study design early in the paper	4
Setting	5	Describe the setting, locations, and relevant dates, including periods of recruitment, exposure, follow-up, and data collection	4
Participants	6	(a) Give the eligibility criteria and the sources and methods of selection of participants	4
Variables	7	Clearly define all outcomes, exposures, predictors, potential confounders, and effect modifiers. Give diagnostic criteria, if applicable	4–5
Data sources/measurement	8*	For each variable of interest, give sources of data and details of methods of assessment (measurement). Describe comparability of assessment methods if there is more than one group	5
Bias	9	Describe any efforts to address potential sources of bias	–
Study size	10	Explain how the study size was arrived at	4–5
Quantitative variables	11	Explain how quantitative variables were handled in the analyses. If applicable, describe which groupings were chosen and why	4–5
Statistical methods	12	(a) Describe all statistical methods, including those used to control for confounding	5
		(b) Describe any methods used to examine subgroups and interactions	5
		(*c*) Explain how missing data were addressed	5
		(*d*) If applicable, describe analytical methods taking account of sampling strategy	5
		(*e*) Describe any sensitivity analyses	5
*Results*			
Participants	13*	(a) Report numbers of individuals at each stage of study—e.g., numbers potentially eligible, examined for eligibility, confirmed eligible, included in the study, completing follow-up, and analyzed	7
		(b) Give reasons for non-participation at each stage	4–5
		(c) Consider use of a flow diagram	–
Descriptive data	14*	(a) Give characteristics of study participants (e.g., demographic, clinical, social) and information on exposures and potential confounders	6
		(b) Indicate number of participants with missing data for each variable of interest	–
Outcome data	15*	Report numbers of outcome events or summary measures	6
Main results	16	(a) Give unadjusted estimates and, if applicable, confounder-adjusted estimates and their precision (e.g., 95% confidence interval). Make clear which confounders were adjusted for and why they were included	6
		(b) Report category boundaries when continuous variables were categorized	6
		(c) If relevant, consider translating estimates of relative risk into absolute risk for a meaningful time period	6
Other analyses	17	Report other analyses done—e.g., analyses of subgroups and interactions, and sensitivity analyses	6
*Discussion*			
Key results	18	Summarize key results with reference to study objectives	7
Limitations	19	Discuss limitations of the study, taking into account sources of potential bias or imprecision. Discuss both direction and magnitude of any potential bias	–
Interpretation	20	Give a cautious overall interpretation of results considering objectives, limitations, multiplicity of analyses, results from similar studies, and other relevant evidence	7–8
Generalizability	21	Discuss the generalizability (external validity) of the study results	7–8
*Other information*			
Funding	22	Give the source of funding and the role of the funders for the present study and, if applicable, for the original study on which the present article is based	09

An Explanation and Elaboration article discusses each checklist item and gives methodological background and published examples of transparent reporting. The STROBE checklist is best used in conjunction with this article (freely available on the Web sites of PLoS Medicine at http://www.plosmedicine.org/, Annals of Internal Medicine at http://www.annals.org/, and Epidemiology at http://www.epidem.com/). Information on the STROBE Initiative is available at http://www.strobe-statement.org

* Give information separately for exposed and unexposed groups

## Abbreviations

BMI: body mass index
CI: confidence interval
