# Supplementary figures and images for: Influence of sexual maturation status on the relationship between body adiposity indicators and age: a cross-sectional study
Source: BMC Res Notes. 2019 Jan 25;12:61. doi: 10.1186/s13104-019-4095-5 (PMC6347827; doi:10.1186/s13104-019-4095-5)

**
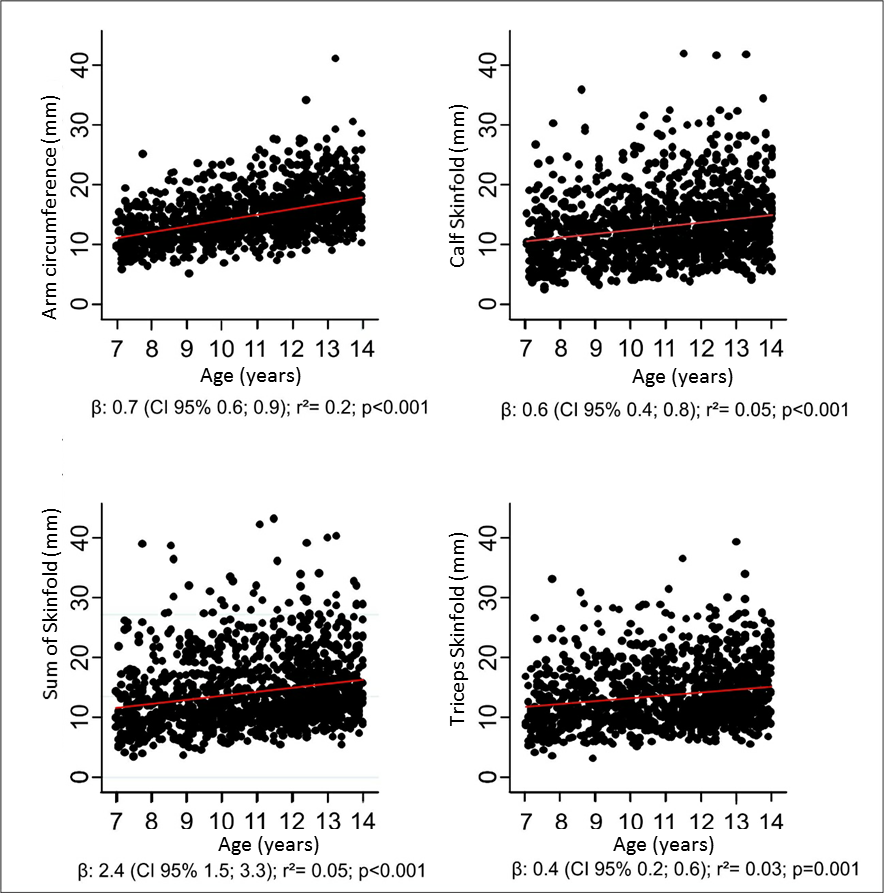
**

Supplement: Supplementary file 1 — Additional file 1: Figure S1. Correlation between age and body adiposity indicators in girls. [file 13104_2019_4095_MOESM1_ESM.docx]
